# Habitat Response Variability: Modeling Predictions Display the Expansion–Contraction Scenario of Two Chinese Endangered Cheirotonus Beetles Under Climate Change

**DOI:** 10.1002/ece3.72156

**Published:** 2025-10-02

**Authors:** Hao Yu, Ya‐gang Shen, Jafir Muhammad, Muzamil Abbas, Yang Cheng, Xia Wan

**Affiliations:** ^1^ Department of Ecology, School of Resources and Environmental Engineering Anhui University Hefei China; ^2^ Anhui Tianma National Nature Reserve Administration Lu'an China

**Keywords:** *Cheirotonus gestroi*, *Cheirotonus jansoni*, China, climate change, habitat sustainability, MaxEnt model, species distribution modeling

## Abstract

Predicting the potential adaptation zones of *Cheirotonus gestroi* Pouillaude and *Cheirotonus jansoni* Jordan under the influence of climate change is essential for understanding their geographical distribution and informing effective species conservation strategies. Species distribution models (SDMs), particularly the MaxEnt model, enable researchers to estimate habitat suitability based on current and future environmental conditions. In this study, we employed the optimized MaxEnt model in combination with ArcGIS software to predict suitable habitats under both present and future climate scenarios (2050s and 2070s), and to identify key environmental variables influencing their geographical distribution. For *C. jansoni*, the influential factors were temperature seasonality (bio4; 31.8%), Elevation (Elev; 28.8%), and precipitation of the driest month (bio14; 24.2%). Currently, its suitable habitats are mainly located in the southeastern part of China, including Zhejiang, Fujian, Hunan, Guizhou, Guangdong, Guangxi, Anhui, Hainan, and Taiwan. Habitat suitability for this species is projected to expand under most climate change scenarios. Conversely, the distribution of *C. gestroi* is primarily shaped by Isothermality (bio3; 68.4%), the Normalized Difference Vegetation Index (NDVI; 19.5%) and Temperature annual range (bio7; 11.7%). This species' suitable habitats are currently concentrated in Yunnan province in southwestern China, with a predicted contraction in habitat range under future climate conditions. The MaxEnt model predictions reveal clear differences in the ecological niches and habitat preferences of these two beetle species, indicating that *C. jansoni* exhibits greater environmental adaptability compared to *C. gestroi* . These findings offer valuable insights for developing targeted monitoring and conservation strategies for these endangered beetles in the face of ongoing climate change.

## Graphical abstract

In this paper, the Maxent model is used to predict two rare insect species of the genus Cheirotonus to understand the impact of future climate change on the suitable habitats of endangered species. The results show that the area of suitable habitats for *Cheirotonus gestroi* will decrease in most future scenarios, and bio3 (Isothermality), bio7 (Temperature annual range) and NDVI (Normalized Difference Vegetation Index) are the main influencing factors; the area of suitable habitats for *Cheirotonus jansoni* will expand, and bio4 (Temperature seasonality), bio10 (Mean temperature of warmest quarter), and bio14 (Precipitation of driest month) are the main influencing factors. The research results can provide theoretical support for the field conservation work of the endangered and rare insects of the genus Cheirotonus.

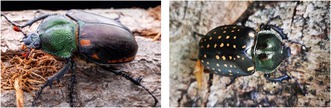

## 1 Introduction

Currently, global climate change is having a dramatic effect on species distributions, particularly affecting poikilothermic insects and their ecosystem (Beaury et al. 2020; Skendžić et al. 2021). Shifts in climatic conditions can lead to changes in habitat range, habitat fragmentation, and accelerated biodiversity loss globally (Ruizhi et al. 2021; Ostad‐Ali‐Askar et al. 2018). The Sixth Assessment Report of the IPCC warns that the impact of climate change'simpact on biodiversity is continuously escalating (Masson‐Delmotte et al. 2021; Jiang et al. 2022), with the average global temperature projected to rise by 0.9°C–4.8°C by the end of the 21st century compared to the levels from 1850 to 1900 (Zhou 2021). Numerous studies have demonstrated that climate change influences species distributions and that future climate conditions will likely affect the potential distribution range of various species (Wang et al. 2020; Li et al. 2020; Zhang et al. 2020). Endangered species are particularly vulnerable to these changes; for instance, for the habitat of *Trachypithecus francoisi* is projected to contract substantially (Wan et al. 2024), while the medium to high suitability habitats for Michelia lacei W.W. Smith are gradually diminishing (Liu et al., 2024). Given the broad impacts of current climate change, understanding species distribution patterns and developing effective conservation strategies based on predicted suitable zones are essential for preserving biodiversity (Williams et al. 2007).

Species distribution models (SDMs) are critically important in macroecology as they are essential tools for forecasting how species may respond to climate change (Iverson and McKenzie 2013; Murphy and Smith 2021). By integrating species occurrence data with environmental variables, SDMs can estimate the potential geographical distribution of species (Elith and Leathwick 2009). Among many modeling techniques, MaxEnt has become a prominent tool, using species distribution data and environmental layers to apply machine learning algorithms based on the principle of maximum entropy, providing reliable predictions of species distribution (Elith et al. 2006). In contrast to other algorithms to characterize species niches, MaxEnt yields more precise predictions (Zhao et al. 2024; Feng et al. 2019). It is especially known for its simplicity and ability to function effectively with limited distribution data, offering high predictive accuracy without requiring extensive fieldwork (Elith et al. 2006; Merow et al. 2013). MaxEnt has been widely used to forecast potential habitats for various species, with applications extending to the control of invasive species and the conservation of endangered species (Wang et al. 2024; Wang, Lu, et al. 2023). Furthermore, the application of the ENMtools package to optimize the MaxEnt settings model effectively diminishes its complexity and minimizes the risk of overfitting (Kass et al. 2021). This is achieved by eliminating highly correlated environmental variables, thereby improving model performance and ensuring more reliable predictions (Wang, Lu, et al. 2023).

**FIGURE 1:**
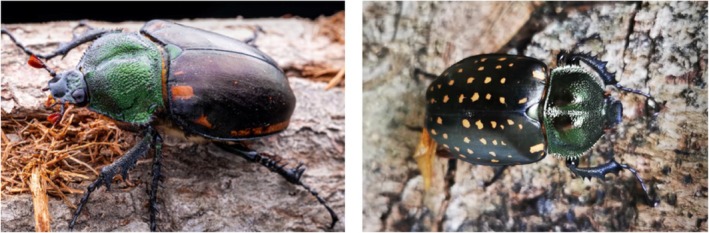
Ecological photographs of *Cheirotonus jansoni* (left) and *Cheirotonus gestroi* (right) in their natural habitats. *Cheirotonus jansoni* photographed by Feng Zhenhao and *C. gestroi* photographed by “Yecai Xiaoxuesheng”.

Species of the genus Cheirotonus are large‐bodied, morphologically distinct beetles with high ornamental value and low population density. Their larvae feed on decaying wood, making them ecologically significant (Yi et al. 2015). *Cheirotonus gestroi* and *Cheirotonus jansoni* (Coleoptera: Euchiridae) are two endangered species found in southwestern regions of China, threatened by illegal hunting and habitat fragmentation (Yang et al. 2020a, 2020b). With the ongoing progression of global warming, it is expected that the habitat range for these species will further contract. Members of the Cheirotonus genus exhibit a striking metallic coloration. Notably, *C. gestroi* has distinct dorsal spots, whereas *C. jansoni* lacks such markings (Figure 1). Due to breeding challenges and rarity, both species are listed as Endangered (EN) on the IUCN Red List and are also nationally protected wild animals in China (IUCN [International Union for Conservation of Nature] 2019; Zhu et al. 2023). Although previous studies have shown that climate and environmental factors influence their population size (Huang et al. 2024), research on these species remains limited. Therefore, it is crucial for informing conservation efforts and maintaining biodiversity.

This study focuses on two endangered species within the genus Cheirotonus, aiming to identify the key environmental variables influencing their distribution. It also seeks to propose appropriate conservation strategies in response to climate change. The MaxEnt model was applied to simulate their potential suitable habitats under current and future climate scenarios. ArcGIS software was used for spatial visualization, and the dominant environmental factors were analyzed. Based on the species' projected responses of different species to climate change, suitable conservation measures are proposed to provide theoretical guidance and support for their protection, thereby raising broader public awareness of insect conservation.

## 2 Materials and Methods

### 2.1 Data Collection and Processing

**FIGURE 2:**
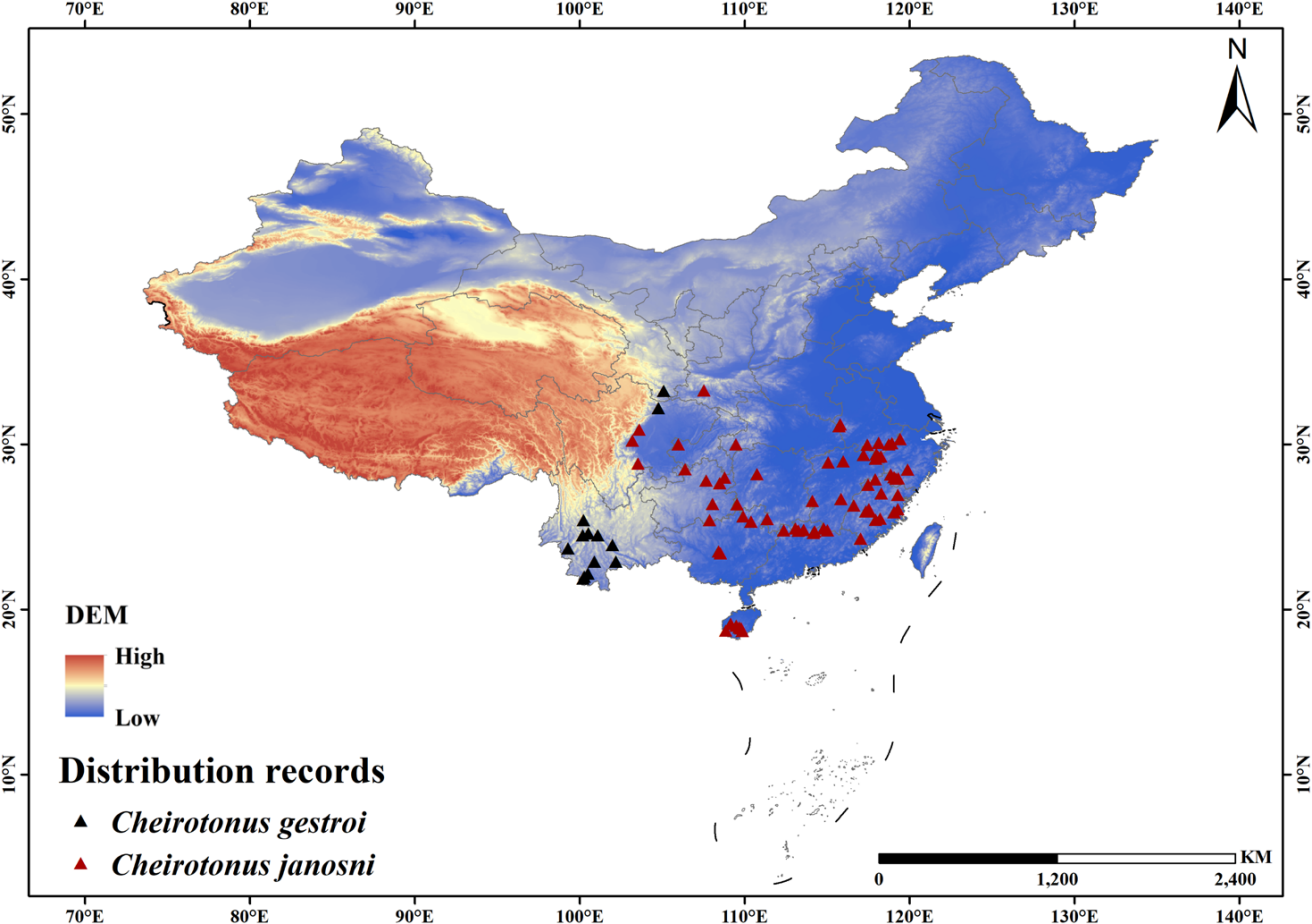
Geographic distribution points of *Cheirotonus gestroi* and *Cheirotonus jansoni* based on occurrence records. The map is based on the State Bureau of Surveying and Mapping Map No. GS(2024)0650 downloaded from the Standard Map Service website. The base map has not been modified. This applies to all maps in the manuscript.

Distribution data for *C. gestroi* and *C. jansoni* were collected from the Global Biodiversity Information Facility (GBIF) database based on human observations (https://www.gbif.org/), supplemented by news reports and field location information. For records lacking precise geographical coordinates, latitude and longitude were approximated using Google Maps, with an estimated accuracy of 10 m. After removing duplicate coordinate entries, there were 22 valid distribution records for *C. gestroi* and 117 valid distribution records for *C. jansoni* . To reduce the impact of the spatial autocorrelation problem caused by closely located occurrence points, only one record was retained within each 10 km × 10 km grid cell (Elith et al. 2011). The SDMtools toolbox (www.sdmtoolbox.org, SDMtoolbox v2.6) in ArcGIS software was used to further refine and optimize the distribution points (Brown et al. 2017). Ultimately, 13 valid occurrence points for *C. gestroi* and 67 valid distribution points for *C. jansoni* were selected (Figure 2). The final dataset was exposed in CSV format for use in MaxEnt model construction (Wang, Duan, et al. 2023).

### 2.2 Acquisition and Screening of Environmental Data

In this study, we obtained 19 bioclimatic variables and 3 topographic factors for current (1970–2000) and future (2050s: 2040–2060; 2070s: 2060–2080) from the WorldClim database (http://worldclim.org) (Balaji et al. 2018). We also selected the Normalized Difference Vegetation Index (NDVI) and Net Primary Productivity (NPP) as vegetation factors (https://www.resdc.cn). All environmental variables were converted to ASCII format using ArcGIS 10.8. For future climate predictions, we utilized the BCC_CSM2_MR dataset (https://worldclim.org), which is known to effectively simulate climate variability across different time scales (Wu et al. 2019). This approach provides a deeper understanding of the potential impact of environmental factors on species distribution. The future climate scenarios were based on four shared socio‐economic pathways (SSPs) (Table 4) (Van Vuuren et al. 2012), with a data resolution of 2.5 (approximately 4.5 km × 4.5 km). Additionally, radiation intensity was positively correlated with the future climate temperature (Shi et al. 2023).

To evaluate the contribution rate of different environmental factors in model training, we conducted a correlation analysis using EMSTools, removing variables with correlation coefficients exceeding ±0.8 and those with low contribution rates (Warren et al. 2021). This step was crucial to ensure the accuracy of the model and to avoid overfitting caused by multicollinearity among environmental variables (Yang et al. 2013). Ultimately, 12 environmental variables were selected for model construction (Table 1). For *C. gestroi* , the chosen variables included bio3, bio7, Slope, and NDVI (Table 2), while *C. jansoni* was associated with bio4, bio5, bio8, bio10, bio14, Slope, and Elevation (Table 3).

**TABLE 1:** Description of environmental variables used in the MaxEnt model to predict the potential distribution of *Cheirotonus gestroi* and *Cheirotonus jansoni*. The variables include 19 bioclimatic parameters, as well as topographic and vegetation indices, expressed in their respective units.

Variables	Description	Units
bio1	Annual mean temperature	°C
bio2	Mean diurnal range	°C
**bio3**	**Isothermality (bio2/bio7) (×100)**	**%**
**bio4**	**Temperature seasonality (standard deviation ×100)**	**%**
**bio5**	**Max temperature of warmest month**	**°C**
bio6	Minimum temperature of coldest month	°C
**bio7**	**Temperature annual range**	**°C**
**bio8**	**Mean temperature of wettest quarter**	**°C**
bio9	Mean temperature of driest quarter	°C
**bio10**	**Mean temperature of warmest quarter**	**°C**
bio11	Mean temperature of coldest quarter	°C
bio12	Annual precipitation	mm
bio13	Precipitation of the wettest month	mm
**bio14**	**Precipitation of driest month**	**mm**
bio15	Precipitation seasonality (coefficient of variation)	**°**
**bio16**	**Precipitation of wettest quarter**	**mm**
bio17	Precipitation of driest quarter	mm
**bio18**	**Precipitation of warmest quarter**	**mm**
bio19	Precipitation of coldest quarter	mm
Aspect	Aspect	**°**
**Slope**	**Slope**	**°**
**Ele**	**Elevation**	**m**
**NDVI**	**Normalized Difference Vegetation Index**	**%**
NPP	Net Primary Productivity	gC/m^2^

*Note:* Variables in bold indicate the bioclimatic factors selected for model construction after correlation analysis and variable screening.

**TABLE 2:** Contribution and permutation importance of environmental variables influencing the distribution of *Cheirotonus gestroi* in the MaxEnt model.

Index	Description	Percent contribution (%)	Permutation importance (%)
bio3	Isothermality (bio2/bio7) (×100)	68.4	67
NDVI	Normalized Difference Vegetation Index	19.5	12.6
bio7	Temperature annual range	11.7	19.8
Slope	Slope	0.4	0.6

**TABLE 3:** Contribution and permutation importance of the environmental variables influencing the distribution of *Cheirotonus jansoni* in the MaxEnt model.

Index	Description	Percent contribution (%)	Permutation importance (%)
bio4	Temperature seasonality (standard deviation × 100)	31.8	23.9
bio5	Max temperature of warmest month	28.8	57.8
bio8	Mean temperature of wettest quarter	24.2	5.9
bio10	Mean temperature of warmest quarter	9.7	7.5
bio14	Precipitation of driest month	4.9	1.0
Slope	Slope	0.3	1.7
Ele	Elevation	0.1	2.3

**TABLE 4:** Description of the four climate change emission scenarios used in future habitat projections.

Scenario	Description
SSP126	A sustainable path for low radiative forcing with a stable radiative forcing of 2.6 W/m^2^ in 2100
SSP245	Intermediate development path of moderate radiative forcing, stable at 4.5 W/m^2^ in 2100
SSP370	Regional competitive development path, radiative forcing stabilizes at 7.0 W/m^2^ in 2100
SSP585	The conventional development path under high radiative forcing stabilizes at 8.5 W/m^2^ in 2100

Abbreviation: SSP, shared socioeconomic pathways.

**TABLE 5:** Evaluation metrics of MaxEnt model performance for *Cheirotonus gestroi* and *Cheirotonus jansoni* based on ENMTools analysis under default and optimized parameter settings.

Parameter settings	RM	FC	ΔAICc	Avg.diff.AUC	Mean.OR_10_
*Cheirotonus gestroi*					
Default	1	LQHP	13.164	0.070	0.158
Optimized	1.5	L	0	0.025	0.105
*Cheirotonus jansoni*					
Default	1	LQHP	105.709	0.033	0.149
Optimized	1	LQ	0	0.037	0.134

*Note:* LQPH: (Limear features, L) + (Quadratic features, Q) + (Product features, P) + (Himgefeatures, H); LQ: (Linear features, L) + (Quadratic features, Q); L: (Quadratic features, Q).

**TABLE 6:** Area under the curve (AUC) values for MaxEnt model predictions of *Cheirotonus gestroi* and *Cheirotonus jansoni* under current and future climate scenarios (2050s and 2070s) based on four Shared Socioeconomic Pathways (SSPs).

Current	2050s				2070s			
	SSP126	SSP245	SSP370	SSP585	SSP126	SSP245	SSP370	SSP585
*Cheirotonus gestroi*								
0.965	0.969	0.958	0.967	0.977	0.954	0.974	0.975	0.977
*Cheirotonus jansoni*								
0.963	0.955	0.958	0.955	0.961	0.954	0.960	0.954	0.959

**TABLE 7:** Areas of suitable zones of Cheirotonus janosni and *Cheirotonus gestroi* in different periods(measured in /×10^4^ km^2^).

Period	Species	High suitable area	Medium suitable area	Low suitable area	Total suitable area
Current	*C. gestroi*	16.57	28.40	100.18	145.15
	*C. janosni*	27.88	35.94	79.83	143.65
2050s SSP126	*C. gestroi*	14.05	25.77	82.25	122.07
	*C. janosni*	32.20	35.75	78.47	146.42
2050s SSP245	*C. gestroi*	19.09	26.81	89.75	135.65
	*C. janosni*	29.34	45.36	70.71	145.41
2050s SSP370	*C. gestroi*	18.34	29.00	89.49	136.83
	*C. janosni*	34.49	48.65	77.58	160.72
2050s SSP585	*C. gestroi*	11.93	26.73	52.89	91.55
	*C. janosni*	26.80	40.06	74.17	141.03
2070s SSP126	*C. gestroi*	24.53	24.91	97.63	147.07
	*C. janosni*	35.46	50.88	81.88	168.22
2070s SSP245	*C. gestroi*	14.58	27.34	71.11	113.03
	*C. janosni*	32.65	44.59	75.34	152.58
2070s SSP370	*C. gestroi*	10.83	26.19	55.83	92.85
	*C. janosni*	31.40	49.20	69.82	150.42
2070s SSP585	*C. gestroi*	13.31	28.11	79.06	120.48
	*C. janosni*	30.98	48.21	74.77	153.96

**TABLE 8:** Changes in the centroid of potentially suitable habitat for *Cheirotonus gestroi* and *Cheirotonus janosni* under current and future climatic scenarios (2050s and 2070s).

Current	2050s				2070s			
	SSP126	SSP245	SSP370	SSP585	SSP126	SSP245	SSP370	SSP585
*C. gestroi*								
100.18° E 27.60° N	101.75° E 26.64° N	101.09° E 27.50° N	100.55° E 27.72° N	102.08° E 26.63° N	100.40° E 27.88° N	101.11° E 27.11° N	102.07° E 26.93° N	99.97° E 27.51° N
*C. jansoni*								
110.53° E 26.81° N	108.40° E 26.85° N	109.12° E 26.54° N	108.97° E 26.85° N	109.57° E 26.87° N	107.71° E 26.66° N	109.01° E 26.94° N	108.48° E 26.92° N	109.22° E 26.87° N

### 2.3 Optimization and Evaluation of the MaxEnt Model

In this study, the ENMeval package in R 4.3.2 was used to optimize the MaxEnt model based on species occurrence points and environmental data (Muscarella et al. 2014). Cross‐validation was conducted using different combinations of regularization multiplier (RM) and feature combinations (FC) to address potential overfitting issues (Muscarella et al. 2014; Phillips and Dudík 2008). RM values ranging from 0.5 to 4 (in 0.5 increments) and eight FC combinations were tested: L, LQ, LQH, LQHP, LQHPT, QHP, QHPT, and HPT, to select the optimal model based on the lowest corrected Delta.AICc score (Kass et al. 2021; Muscarella et al. 2014; Phillips et al. 2017). In addition, the difference between the training and testing AUC (AUC.DIFF) and the 10% training omission rate (OR10) served as supplementary metrics to evaluate the model's goodness‐of‐fit and complexity relative to species distribution (Wang, Duan, et al. 2023).

The species occurrence point data and environmental variables were imported into the MaxEnt software. The model parameters were configured such that 25% of the distribution points were randomly selected as the test set, while the remaining 75% served as the training set. We conducted 10 replicated runs using the Bootstrap method to analyze the predominant environmental factors influencing the distribution of Cheirotonus species. During the modeling, the response curve, jackknife test, and random seed settings were enabled, while all other parameters were left at their default values (Elith et al. 2011; Janitza et al. 2013). Environmental variables were adjusted according to different periods to simulate potential future distributions of suitable habitat areas. In this study, model performance was evaluated using the area under the receiver operating characteristic curve (AUC) (Liu et al. 2023). An AUC value below 0.5 indicates that the model performance is no better than random guessing, while a value above 0.9 reflects high predictive power. The closer the AUC value is to 1, the greater the accuracy of the prediction (Janitza et al. 2013).

### 2.4 Source of Vector Diagram and Software Models

The administrative boundary map of China was obtained from the National Basic Geographic Information System (http://nfgis.nsdi.gov.cn), with a scale of 1:100,000 and map plan no. (2024) 0650. All spatial analyses were conducted using ArcGIS software version 10.8. For species distribution modeling, we employed the Maxent version 3.4.4, which was downloaded from the official source (http://biodiversityinformatics.amnh.org/open/Maxent).

### 2.5 Classification of Suitable Zones and Centroid Shifts Analysis

The prediction results from the MaxEnt model were expressed as habitat suitability values, which were categorized into four levels using Jenks natural breaks classification via the Reclassify tool in ArcGIS 10.8 software (Zhang et al. 2020). In ArcGIS, color gradients were used to indicate habitat suitability levels: white for unsuitable areas, yellow for low suitability, orange for medium suitability, and red for high suitability.

To analyze the spatial shifts in suitable habitat for Cheirotonus species under current and future scenarios, ArcGIS was used to quantify the area and proportion of the suitability category. The predictions for each period were then converted into binary distribution maps. By integrating the SDMTools package, we performed a detailed analysis of habitat range dynamics, identifying expansion, contraction, and stable zones within the predicted suitable ranges. Additionally, centroid shifts of suitable habitats were tracked over time. This enabled the visualization of dynamic suitability changes and the mapping of centroid migration paths for *C. gestroi* and *C. jansoni*.

## 3 Results

### 3.1 Evaluating Model Performance

The optimized MaxEnt model efficiently reduced model complexity (Table 5), thereby mitigating overfitting and improving prediction accuracy (Pica et al. 2024). Based on these optimization results, the model parameters were set to RM = 1.5 and FC = L for *C. gestroi* , and RM = 1 and FC = LQ for *C. jansoni* to ensure the accuracy and robustness of the predictions (Table 6).

**FIGURE 3:**
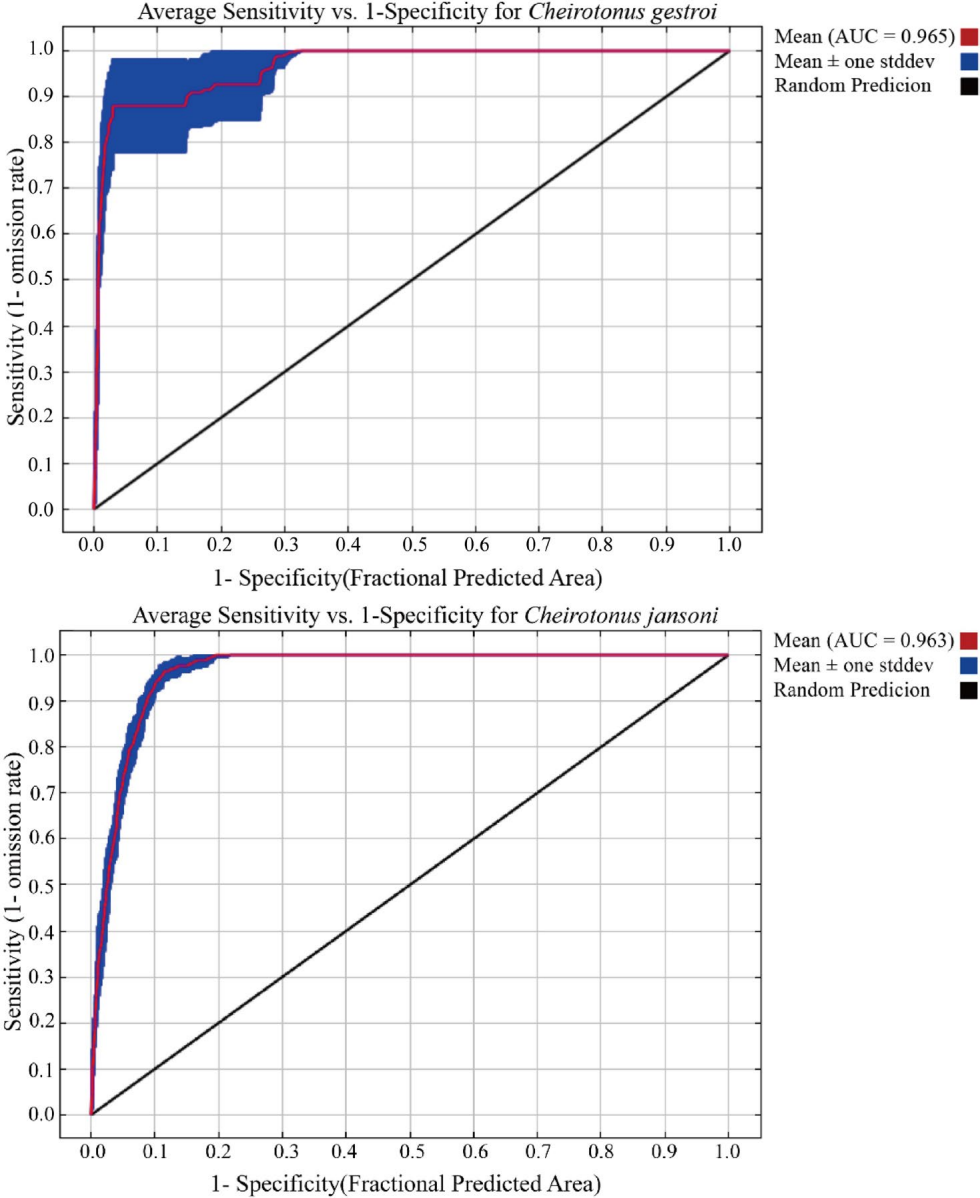
Receiver operating characteristic (ROC) curve for *Cheirotonus gestroi* and *Cheirotonus jansoni* under the current climate conditions, illustrating MaxEnt model performance.

Based on the simulation results of the MaxEnt model (Figure 3), we found that the AUC values corresponding to the ROC curves of *C. gestroi* and *C. jansoni* were both above 0.9–0.965 and 0.963, respectively, indicating the high predictive accuracy of the MaxEnt model in estimating the potential suitable habitats of these species.

### 3.2 Comprehensive Analysis of Environment Variables

**FIGURE 4:**
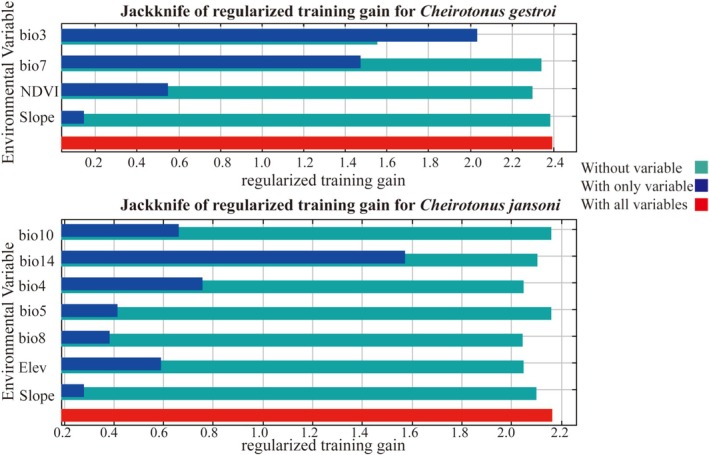
Jackknife test of variable importance for the MaxEnt model, showing the contribution of each environmental variable to the distribution of *Cheirotonus gestroi* and *Cheirotonus jansoni*.

The results of the jackknife test revealed the relative contributions of different environmental variables (Figure 4), enabling a deeper investigation into key factors influencing the geographical distribution of the species (Wang, Duan, et al. 2023). The analysis showed that Isothermality (bio3), Temperature Annual Range (bio7) and Normalized Difference Vegetation Index (NDVI) were the most important predictors of the suitable habitats for *C. gestroi* . Similarly, Precipitation of the Driest Month (bio14) and Temperature Seasonality (bio4) were identified as the most influential environmental factors affecting the distribution of *C. jansoni* , followed by Mean Temperature of the Warmest Quarter (bio10), Elevation (Ele), Maximum Temperature of the Warmest Month (bio5), Mean Temperature of the Wettest Quarter (bio8), and Slope.

**FIGURE 5:**
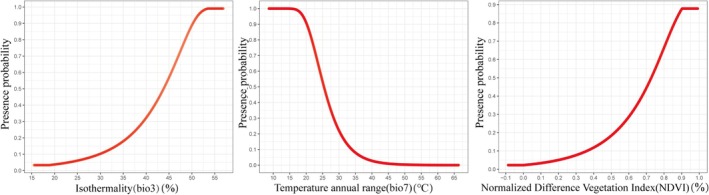
Response curves for *Cheirotonus gestroi* showing the relationship between environmental variables and habitat suitability.

The relationship between the presence probability of *C. gestroi* and environmental variables is shown in Figure 5. In general, areas with a probability value exceeding 0.5 are considered suitable survival habitats for the species. The combined contribution of the top three environmental variables exceeds 90. The results indicate that, within a certain range, the probability of species occurrence increases with Isothermality (bio3) and Normalized Difference Vegetation Index (NDVI), and decreases with Temperature annual range (bio7). Optimal ranges for these environmental variables are as follows: Isothermality (bio3): 43.85%–56.92%; Temperature annual range (bio7): 8.66°C–25.39°C; Normalized Difference Vegetation Index (NDVI): 0.73%–0.99%.

**FIGURE 6:**
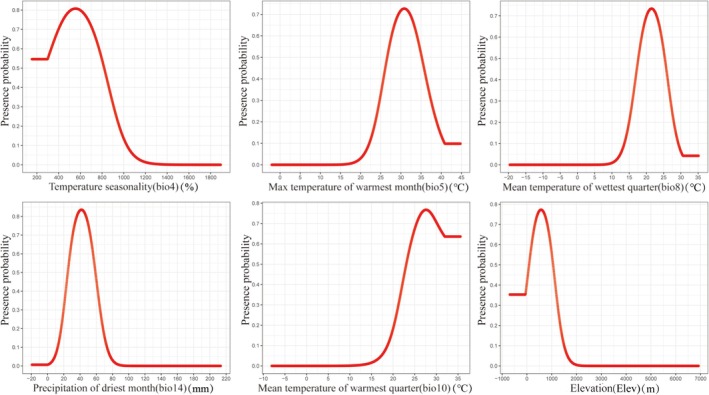
Response curves for *Cheirotonus janosni* illustrating the relationship between habitat suitability environmental variables.

As shown in Figure 6, the presence probability of *C. janosni* initially increased with each environmental variable before subsequently declining. The combined contribution of the top six environmental factors exceeds 90%. The suitable range of Temperature seasonality (bio4) is 153.65%–819.81%; the Max temperature of warmest month (bio5) is 26.60°C–35.13°C; the suitable range of Mean temperature of wettest quarter (bio8) is 17.67°C–25.34°C; Mean temperature of warmest quarter (bio10) is 23.05°C–35.58°C; the suitable range of Precipitation of driest month (bio14) is 25.06–58.82 mm; and the suitable range of Elevation (Ele) is 92.74–1052.11 m.

### 3.3 Prediction of Suitable Areas Under the Current Climate Change Scenario

**FIGURE 7:**
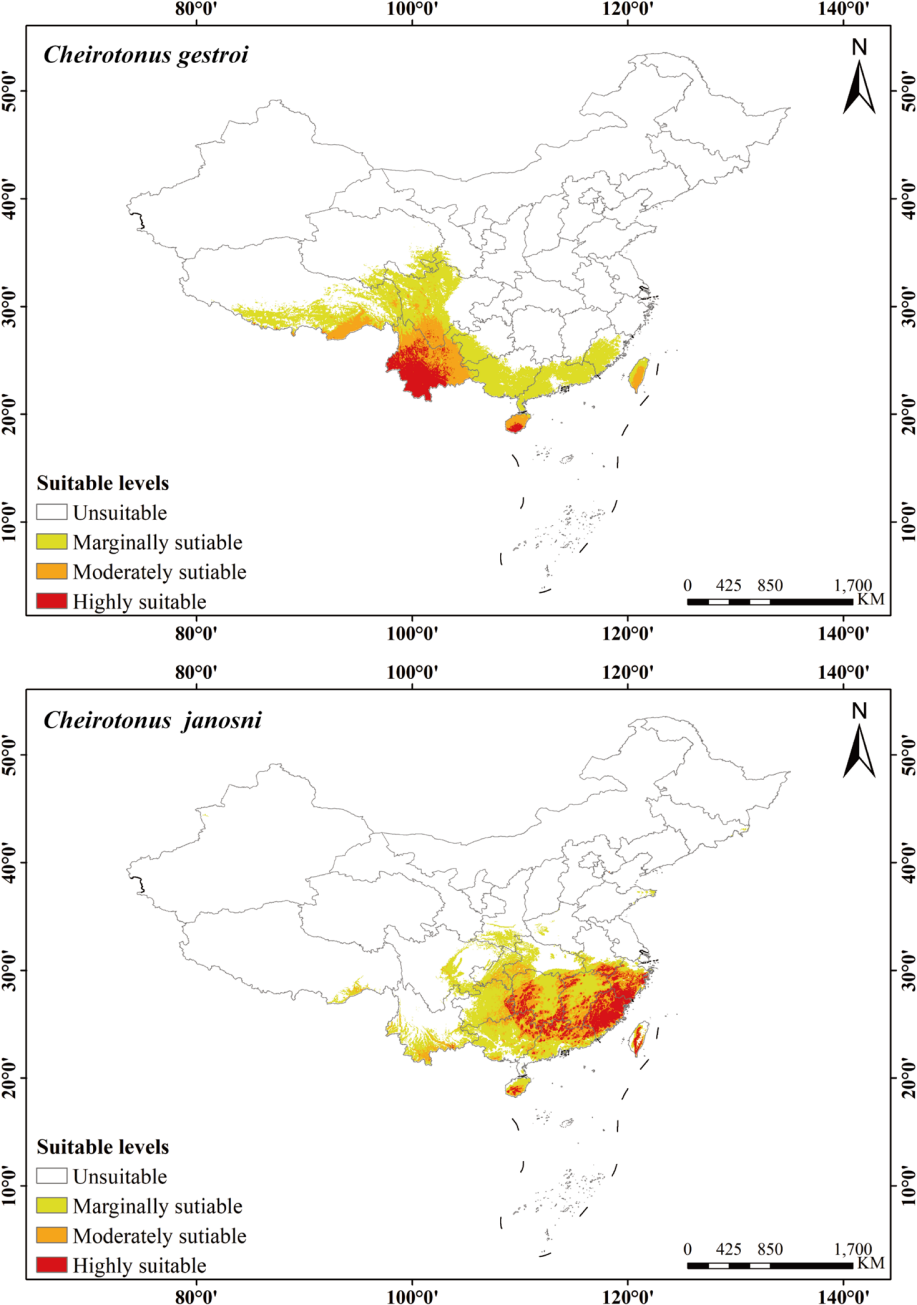
Potential suitable areas of *Cheirotonus gestroi* and *Cheirotonus jansoni* under current climatic conditions. (A) *Cheirotonus gestroi* and (B) *Cheirotonus janosni*.

**FIGURE 8:**
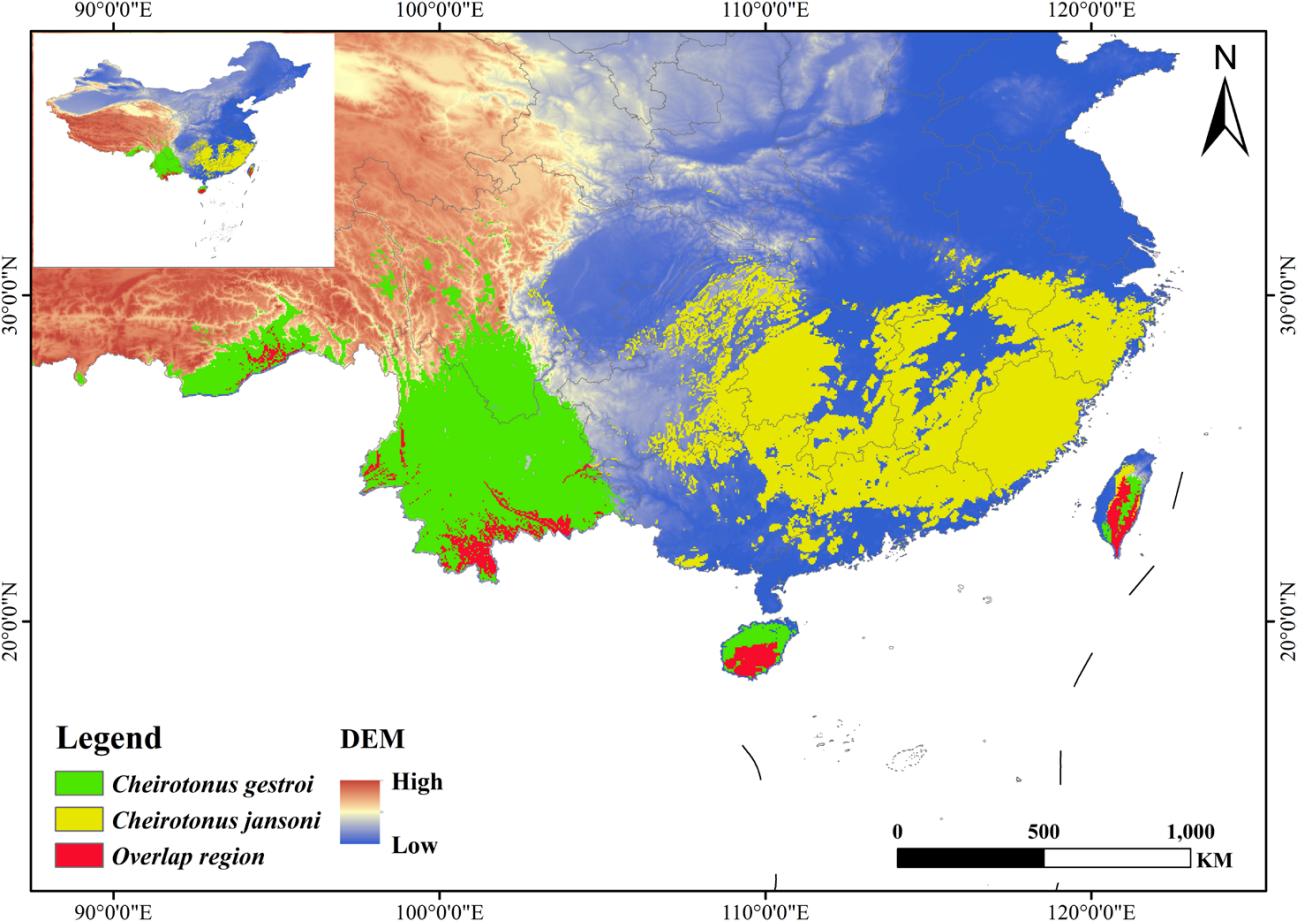
Overlapped potential distribution area of *Cheirotonus gestroi* and *Cheirotonus jansoni* under current climatic conditions. (A) Green: *Cheirotonus gestroi*, (B) Yellow: *Cheirotonus janosni*, and (C) Red: Overlap region.

According to the prediction results from the MaxEnt model, the total area of the suitable habitat for *C. gestroi* is estimated to be 145.15 × 10^4^ km^2^. Within this, high suitability areas occupy 16.57 × 10^4^ km^2^, medium suitability areas cover 28.40 × 10^4^ km^2^, and low areas account for 100.18 × 10^4^ km^2^, representing 1.72%, 2.95%, and 10.40% of China's total area respectively. The suitable habitat for *C. gestroi* is mainly concentrated in Yunnan, Guangdong, Guangxi, Fujian, Sichuan, Tibet, Hainan, Taiwan, and adjacent areas. High suitability areas are primarily found in central and southern Yunnan and southern Hainan, while medium suitability areas are mainly distributed in northern Yunnan, southern Sichuan, central and northern Hainan, and southern Tibet (Figure 7; Table 5). The total suitable habitat area of *C. janosni* is estimated to be 143.65 × 10^4^ km^2^. Among them, the high suitability areas comprise 27.88 × 10^4^ km^2^, medium suitability areas cover 35.94 × 10^4^ km^2^, and low suitability areas span 79.83 × 10^4^ km^2^, accounting for 2.89%, 3.73%, and 8.29% of the total area of China's total area. The suitable habitat for *C. janosni* is mainly concentrated in Guangdong, Guangxi, Fujian, Zhejiang, Guizhou, Hunan, Jiangxi, Chongqing, Anhui, Hainan, and Taiwan provinces and their surrounding areas. High suitability zones are predominantly found in most areas of Fujian, Zhejiang, Guangdong, Guangxi, Hunan, Jiangxi, and Guizhou provinces and in parts of Anhui, Hainan, and Taiwan, while medium suitability areas are mostly distributed on the margins of high suitability zones (Figure 7; Table 5). At the current stage, the overlap between the two species' suitable areas is rather limited, corresponding to their actual known sites. The suitable habitat ranges of the two species differ significantly, with only a partial overlap in Hainan, Taiwan, Yunnan provinces, and Tibet (Figure 8).

### 3.4 Changes in the Area of Suitable Areas Under Future Climate Scenarios

**FIGURE 9:**
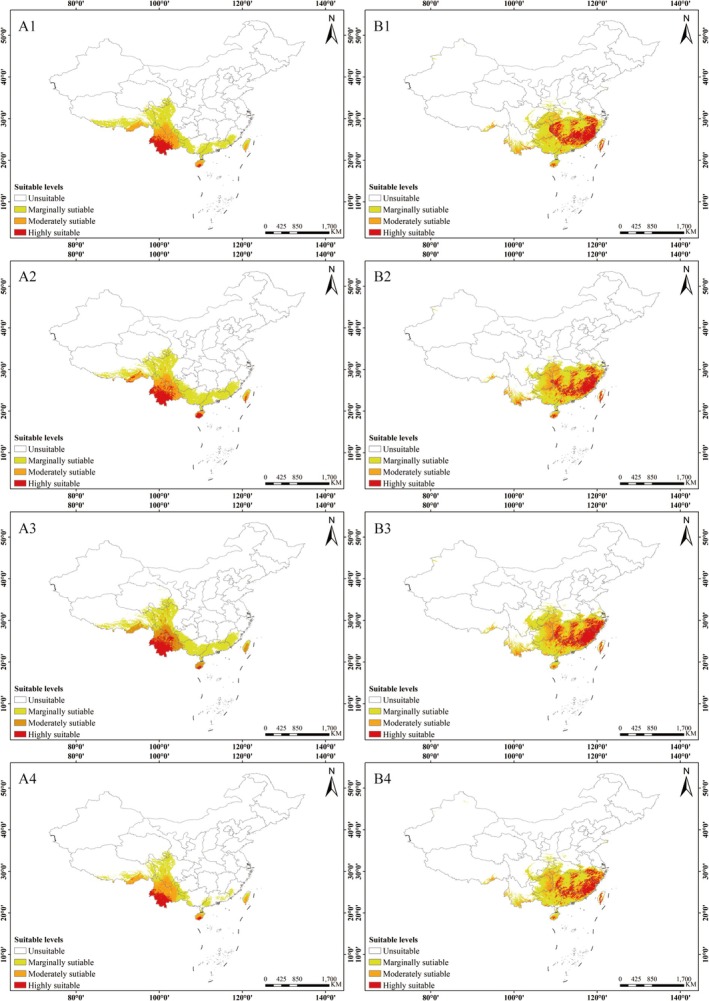
Prediction of potential suitable areas for *Cheirotonus gestroi* and *Cheirotonus jansoni* in China for the 2050s under different emission scenarios. *Cheirotonus gestroi*: (A1) 2050s SSP126; (A2) 2050s SSP245; (A3) 2050s SSP370; (A4) 2050s SSP585. (B) *Cheirotonus janosni*: (B1) 2050s SSP126; (B2) 2050s SSP245; (B3) 2050s SSP370; (B4) 2050s SSP585.

**FIGURE 10:**
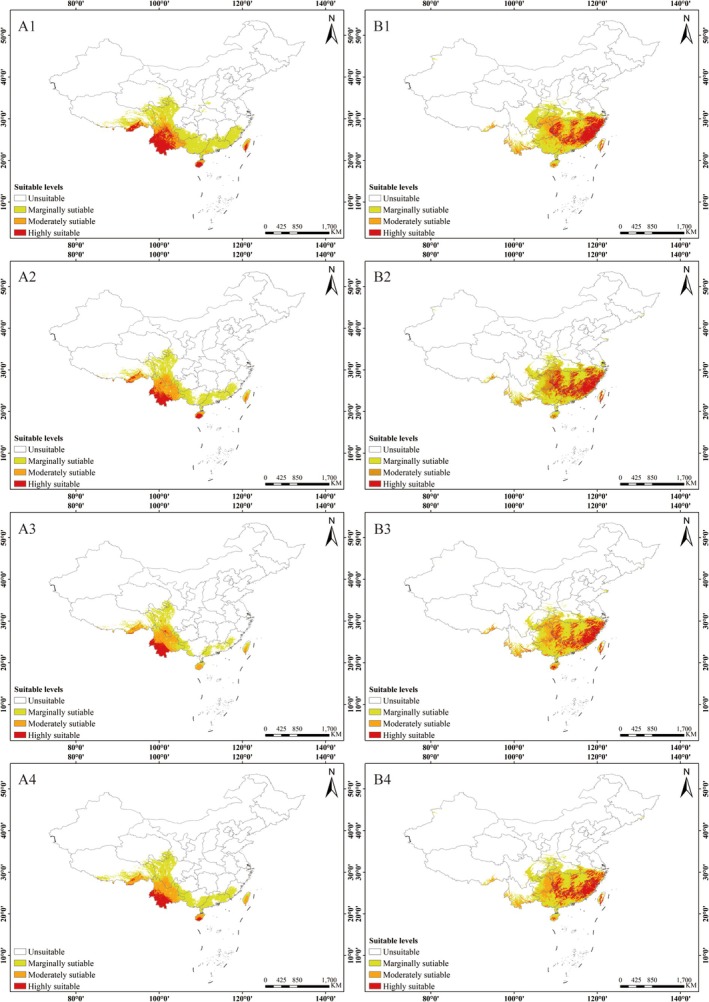
Prediction of potential suitable areas for *Cheirotonus gestroi* and *Cheirotonus jansoni* in China for the 2070s under different emission scenarios. (A) *Cheirotonus gestroi*: (A1) 2070s SSP126; (A2) 2070s SSP245; (A3) 2070s SSP370; (A4) 2070s SSP585. (B) *Cheirotonus janosni*: (B1) 2070s SSP126; (B2) 2070s SSP245; (B): 2070s SSP370; (B4): 2070s SSP585.

In this study, we selected two different future periods, the 2050s and 2070s, to predict the potential distribution patterns of *C. gestroi* and *C. janosni* under various climate change scenarios (Figures 9 and 10; Table 7). Overall, the suitable habitat area for *C. gestroi* is projected to decline, with the high suitability area shrinking to its smallest size of only 10.83 × 10^4^ km^2^ in the 2070s under the SSP370 scenario. In most scenarios, the medium suitability zone shows a slight reduction, reaching its maximum in the 2050s under the SSP370 scenario.

Except under the 2050sSSP585 scenario, the overall suitable living area of *C. janosni* in the future is smaller than that of the present, while the suitable area in other scenarios has increased. The high suitability zone for *C. jansoni* expanded in most scenarios, reaching its maximum in 2070sSSP126 scenario. The total suitable area also increased, peaking under the 2070sSSP126 scenario.

### 3.5 Centroid Shifts in Potential Distribution

**FIGURE 11:**
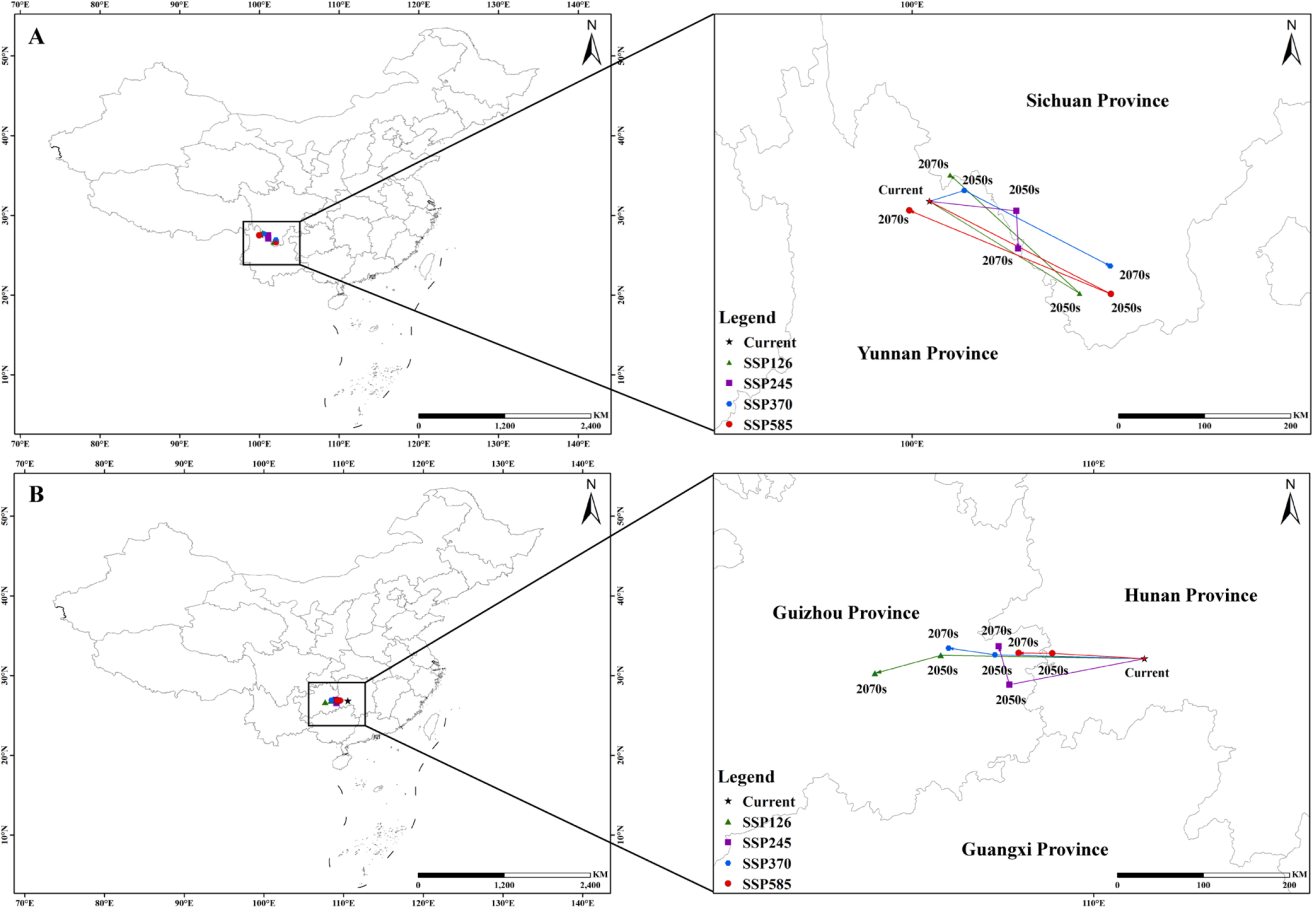
Changes in the centroid of the potential suitable distribution for *Cheirotonus gestroi* and *Cheirotonus jansoni* under current and future climatic scenarios. (A) *Cheirotonus gestroi*; (B) *Cheirotonus janosni*.

As shown in Figure 11 and Table 8, the centroid displacement of *C. gestroi* shows varying shifts under different scenarios, but it generally tends to move southeast in general. Under the SSP126 and SSP585 scenarios, the distribution center shifts southeast first and then northwest; under the SSP245 scenario, the centroid shifts eastward first and then to the south; under the SSP370 scenario, the centroid shifts eastward and then to the north. In general, the centroid shift of *C. janosni* shifts westward. Specifically, under the centroid shift of SSP126, SSP370, and SSP585 scenarios, the centroid consistently shifts westward, while under the SSP245 scenario, it shifts westward first and then northward.

## 4 Discussion

The potential distribution of this species is predominantly situated within the central‐southern and eastern region. These regions are characterized by high levels of biodiversity, complex and heterogeneous ecological settings, and climatic patterns marked by elevated temperatures and substantial rainfall (Zhi et al. 2021). Such environmental attributes collectively render these areas highly conducive to the survival and proliferation of beetles (Zhi et al. 2021). This study represents the first application of the MaxEnt model to dissect the spatial distribution configuration of Cheirotonus beetles and to evaluate the influence of climate change upon their potentially suitable habitats. By utilizing the ENMTools package to refine the MaxEnt model, we can effectively reduce its complexity and lower the risk of overfitting (Muscarella et al. 2014). This approach involves eliminating highly collinear environmental variables, thereby enhancing the model's accuracy and ensuring more reliable predictions (Feng et al. 2019). In this study, the MaxEnt model and ArcGIS software were used to represent the current and future potential distribution of *C. gestroi* and *C. janosni*, yielding highly reliable prediction results, with AUC values of 0.965 and 0.963, which can serve as a reference for the potential geographic distribution.

Under current climatic conditions, the highly suitable distribution area for *C. gestroi* is primarily concentrated in southwest China, including the provinces of Yunnan and Hainan which is highly correlated with the actual distribution points. The warm and moist conditions are especially conducive to their survival. Additional research has reinforced this finding (Yi et al. 2015; Yang et al. 2020a, 2020b). Compared with the current climate, the centroid of *C. gestroi* in the future shows a tendency to migrate south. This shift to higher altitudes and latitudes is a trend observed by many species due to global warming (Prevéy et al. 2020). Under the 2050s SSP585 and 2070s SSP370 scenarios, the high‐suitability areas and total suitable area of *C. gestroi* contracted sharply, signifying that the influence of climate warming on this species is highly substantial. Similarly, under diverse climate scenarios, Cedrus atlantica is at a high risk of extinction (Laala and Adimi 2024); Eversmannia subspinosa's shows a substantial reduction in high‐probability habitats by 2060 (Zaheri et al. 2024). Temperature is the main environmental factor influencing the potential distribution of beetles (Kistner‐Thomas 2019; Aidoo et al. 2022) This implies that species possessing a narrow ecological niche may encounter greater challenges in coping with the menace of climate warming. The jackknife method elucidates that the distribution of *C. gestroi* is minimally affected by slope and mainly affected by Isothermality (bio3, 68.9%) and Normalized Difference Vegetation Index (NDVI, 19.5%), while it decreases with Temperature annual range (bio7, 11.7%), which aligns with its habitat characteristics. The response curve showed that the presence probability of *C. gestroi* is the highest when Isothermality (bio3) is between 53.52% and 56.92%, Temperature annual range (bio7) is between 8.66°C and 13.39°C, and Normalized Difference Vegetation Index (NDVI) is between 0.90 and 0.99. Relevant studies indicate that bio3 is the most significant environmental variable influencing the potential distribution of Heteronychus arator and Oryctes boas, with their potential distribution areas in China exhibiting similarities to that of *C. gestroi* (Wang, Lu, et al. 2023). The sampling site habitats were primarily defined by mountainous forest vegetation types that exhibit significant vegetation coverage. With the change of climate, the distribution range of *C. gestroi* in most future scenarios will decrease, and the minimum area in the 2050s SSP585 scenario is projected to decrease by 36.9% compared with the contemporary total suitable area, indicating that different emission intensities have a greater impact on the distribution range of species. Overall, the distribution of insects is influenced by various environmental factors, including temperature, rainfall, and elevation (Gao et al. 2023, 2024; Gerber et al. 2023).

*Cheirotonus janosni* is an endangered insect species from Southeast Asia (Shao et al. 2014). Under the current scenario, *C. janosni* is mainly distributed across multiple provinces of southern China with a wide range. Compared with the current distribution, the future centroid will expand westward. Across all future scenarios, the total suitable area of *C. janosni* exhibits an enlargement and attains its peak in the 2070s SSP126, registering an approximate growth of 17.10%. Remarkably, despite *C. janosni* possessing a comparatively wide distribution scope, its highly suitable area still demonstrates a decreasing propensity in the 2050s SSP585 scenario. The main environmental factors affecting *C. janosni* distribution are Temperature seasonality (bio4, 31.8%), Elevation (Ele, 28.8%) and Precipitation of driest month (bio14, 24.2%). The response curve shows that *C. janosni* is most likely to exist when Temperature seasonality (bio4) is approximately 557.17, Elevation (Ele) is around 572.42 m, and Precipitation of driest month (bio14) is approximately 41.59 mm. Research indicates that factors such as Temperature seasonality (bio4), Elevation (Ele), and Precipitation of driest month (bio14) influence the distribution of Lucanidae beetles. In China, most species of Lucanidae are distributed in South China, with the hotspot being southeastern Tibet (Chen et al. 2020). Precipitation may exert indirect influences on the species richness of Lucanus, given that the larvae of these stag beetles inhabit and utilize dead wood as a food source (Songvorawit et al. 2017). In addition, the range of species may expand and shift due to the effects of global warming (Wei et al. 2018). In general, the distribution range of *C. janosni* will further expand in the future and reach its maximum in the 2070s SSP126 scenario.

The presence of species is affected by both biological and abiotic factors, and incorporating biological elements can improve the precision of models that predict species distribution (De Araújo et al. 2014). Moreover, abiotic pollutants—such as microplastics and nanoplastics—have been shown to impair critical insect traits, including survival, development, and reproduction, thereby posing additional threats to species persistence (Abbas et al. 2025). Research has demonstrated that the introduction of mammalian predators in New Zealand exerts selective pressures on populations of the stag beetle Geodorcus helmsi, resulting in shifts in both population demographics and the distribution of phenotypic traits (Grey et al. 2024). This study focuses solely on the influence of non‐biological elements on the distribution of species, without extensively exploring associated biological factors. In future studies, it is crucial to take into account human activities and the influence of other species as factors in models that predict species distribution. Enhanced conservation initiatives are essential in these regions, including the establishment of protected zones and improved measures for population safeguarding to mitigate potential extinction threats.

## 5 Conclusion

This study employed an optimized MaxEnt model to forecast the potentially suitable regions for *C. gestroi* and *C. janosni* in China, considering both present and future climatic scenarios. The findings suggest that the southwestern region currently represents the primary suitable habitat for *C. gestroi* , but this area is expected to diminish over time, with a centroid shifting southward. In contrast, the data indicate that much of southern China serves as a key suitable zone for *C. janosni* at present, and this area is projected to expand in the future, with a trend of centroid shift towards the west. This research provides important perspectives on the conservation of *C. gestroi* and *C. janosni* under both current and anticipated scenarios, which can guide the development of effective protective measures for these species.

## Author Contributions

**Hao Yu:** conceptualization (lead), data curation (lead), formal analysis (lead), investigation (equal), methodology (equal), project administration (equal), resources (equal), software (lead), supervision (equal), validation (lead), visualization (lead), writing – original draft (lead), writing – review and editing (equal). **Ya‐gang Shen:** data curation (equal), investigation (equal), methodology (equal), validation (equal), visualization (equal), writing – review and editing (equal). **Jafir Muhammad:** data curation (equal), formal analysis (equal), investigation (equal), writing – review and editing (equal). **Xia Wan:** conceptualization (equal), data curation (equal), funding acquisition (lead), methodology (equal), project administration (lead), resources (equal), supervision (equal), validation (equal), writing – review and editing (equal). **Muzamil Abbas:** data curation (equal), formal analysis (equal), writing – review and editing (equal). **Yang Cheng:** data curation (equal), investigation (equal), validation (equal).

## Ethics Statement

This manuscript does not include human or animal research. If this manuscript involves research on animals or humans, it is imperative to disclose all approval details.

## Conflicts of Interest

The authors declare no conflicts of interest.

## Data Availability

The data supporting the findings of this study have been deposited in the Dryad Digital Repository (https://datadryad.org/dashboard), under the provisional https://doi.org/10.5061/dryad.1rn8pk14v. The dataset is currently available for peer‐review purposes.
